# A Systematic Review on the Role of the Stria Vascularis in Menière’s Disease Pathogenesis

**DOI:** 10.1007/s10162-025-01006-y

**Published:** 2025-09-23

**Authors:** Pablo Cruz-Granados, Sreeparna Das, Kiana Bagheri-Loftabad, Jose A. Lopez-Escamez

**Affiliations:** 1 Meniere Disease Neuroscience Research Program, Faculty of Medicine & Health, School of Medical Sciences, The Kolling Institute, University of Sydney, Sydney, NSW Australia; 2 Otology & Neurotology Group CTS495, Division of Otolaryngology, Department of Surgery, Instituto de Investigación Biosanitaria, Ibs.GRANADA, Universidad de Granada, Granada, Spain; 3 Sensorineural Pathology Programme, Centro de Investigación Biomédica en Red en Enfermedades Raras, CIBERER, Madrid, Spain; 4 Ear Science Institute Australia, Nedlands, WA Australia

**Keywords:** Menière’s Disease, Stria vascularis, Autoimmune, Inflammation, Hearing loss, Genetics

## Abstract

**Purpose:**

The stria vascularis (SV) is a secretory epithelium that maintains fluid homeostasis and generates the endocochlear potential in the cochlear duct. Multiomic studies have identified genes in the SV that could contribute to the pathogenesis of Menière’s Disease (MD), a disorder defined by episodic vertigo, sensorineural hearing loss, and tinnitus. This systematic review identified genes expressed in the SV cell types (marginal, intermediate, and basal) and gap junction proteins to evaluate their pathophysiological connections to MD.

**Methods:**

We conducted a literature search on 1293 articles relevant to MD and SV that were screened for SV genes involved in MD. Following quality assessment, 130 studies met the inclusion criteria, comprising 26 human studies, 101 animal studies, and three human-animal studies.

**Results:**

Seven immune-related and six auditory-related genes were identified: *CACNA1D*, *ESRRB*, *HGF*, *KCNE1*, *MDH1*, *QSOX1*, and *SLC12A2* (marginal cells); *ACTB*, *TMEM176A*, and *TMEM176B* (intermediate cells); and *ACTN1*, *COL11A2*, and *GSTM1* (basal cells). Gene-set-enrichment-analysis revealed pathways involving gap-junction assembly and electrical coupling. International Mouse Phenotyping Consortium data showed *Gja1* and *Kcne1* knockouts have immune system abnormalities. Single-cell RNA sequencing data of the lateral wall revealed high expression of *Coch*, *Dtna*, and *Prkcb* in fibrocytes, Reisner’s cells, and immune cells. Furthermore, TWEAK released from intermediate cells and bound to its receptor (*TNFRSF12A*) in the marginal cells may upregulate NF-κB inflammatory response in MD patients.

**Conclusion:**

We hypothesize that some SV genes may contribute to the audiovestibular phenotype in MD, but most of them play a role in the altered immune response found in Sporadic MD.

**Supplementary Information:**

The online version contains supplementary material available at 10.1007/s10162-025-01006-y.

## Introduction

Menière’s Disease (MD) is an inner ear syndrome characterized by sensorineural hearing loss (SNHL) with tinnitus and episodes of vertigo of multifactorial origin, with a significant heritability according to familial aggregation and sequencing studies in familial and sporadic cases [1]. Menière’s Disease is prevalent across Europe and East Asia, with a greater number of cases reported in the European population [2]. Several MD clinical subgroups have been described according to the familial history of MD and associated comorbidities, including migraine and autoimmune conditions [3]. Additionally, distinct phenotypes have been identified, according to their cytokine profiles and systemic inflammation, with one group exhibiting higher levels of Th2 cytokines, IgE and a type 2 immune response, and another one with high levels of IL-1β and autoinflammation [4–6].

The accumulation of endolymph fluid in the scala media is associated with the pathophysiology of MD. The endolymphatic hydrops (EH) leads to increased pressure within the cochlear duct with an enlargement of the endolymphatic space, which results in damage to the organ of Corti and other inner ear membranes. However, vertigo attacks, hearing loss, or tinnitus in MD are not explained by EH alone, as individuals without MD have been found with EH [7].

The stria vascularis (SV) is an organ made up of three main cell layers, located in the lateral wall of the cochlear duct [8]. It is surrounded by the spiral ligament and plays a crucial role in regulating K^+^ homeostasis in the scala media and generating the endocochlear potential, which is essential for hearing [9, 10].

Exome sequencing and genome sequencing in SNHL and MD have identified a significant number of genes with a burden of rare variation in differentially expressed genes (DEG) in the SV [11, 12].

This review aims to understand the importance and role of SV genes in the development of MD.

## Methodology

This review was conducted following the Preferred Reporting Items for Systematic Reviews and Meta-Analyses (PRISMA) [13] guidelines for systematic reviews, and it abided by the Meta-analyses Of Observational Studies in Epidemiology (MOOSE) [14] checklist. This review’s protocol was registered on PROSPERO (CRD42024618482).

The PICO (Participants–Intervention–Comparison–Outcomes) question included the following:

- Participants: studies including datasets from individuals with definite Sporadic MD or Familial MD, or MD animal models, or cellular models.
- Intervention: epigenomic, genomic, transcriptomic, or proteomic studies related to the stria vascularis and genes linked to MD.
- Comparison: healthy controls, individuals without MD, or animal models not affected by the disease.
- Main outcome: identification of differentially expressed genes, epigenetic modifications, differentially expressed transcripts, or proteins in the stria vascularis associated with MD onset or progression.
- Secondary outcomes: predicted enriched biological pathways, biomarkers, or mechanisms linked to MD development, particularly relating to endolymphatic hydrops, fluid balance, or inner ear function.
- Study design: case–control studies, cohort studies, animal models, genomic and proteomic analyses of tissue samples, or epigenomic profiling of DNA.

### Search Strategy

The search strategy was carried out on 18th November 2024 using original articles published in the year 2005 onwards from PubMed, Scopus, and Cochrane databases. The MeSH terms used were as follows: *(Meniere OR Stria Vascularis) AND (Epigenomics OR Epigenetics OR Genomics OR Genetics OR Gene or Transcriptomics OR RNAseq OR Protein)*. Duplicate articles and articles not relevant to the review’s objective were excluded. Furthermore, the following exclusion criteria were also used:

- Studies published in other languages other than English
- Single-case reports.

### Data Collection

Two independent reviewers (P.C.G. and S.D.) reviewed all the abstracts to select records according to the inclusion criteria. When consensus could not be reached, a third reviewer (J.A.L.E.) was involved in resolving discrepancies. We summarized the studies that involved epigenetic changes, genes, transcripts, and proteins from different studies on MD and SV.

### Gene Search Using Published Datasets

Further filtering was performed on selected studies to identify those involving genes of interest. DOIs were retrieved, and three independent reviewers (P.C.G., S.D., and K.B.L.) screened the introduction, results, and discussion sections of each study using a tailored list composed of genes associated with Familial MD (*FAM136A*, *DTNA*, *PRKCB*, *COCH*, *DPT*, *SEMA3D*, *TECTA*, *GUSB*, *SLC6A7*, *HMX2*, *LSAMP*, *OTOG*, *STRC*, *MYO7A*, and *GJD3*) [15, 16], marker genes for SV marginal, basal, and intermediate cells from two studies using postnatal day 30 (P30) mice [17, 18] and gap junction proteins (connexins). For SV cell markers, only genes with a ± onefold change (logFC) and > 0.05 *p*-value adjusted were used. The list of tailored genes can be found in Supplementary Table 1. For genes, the Human Genome Organization (HUGO) nomenclature was used, and for proteins only the recommended name was employed. Both gene and protein identifiers were extracted from UniProt [19] to use as search keywords. Studies that replicated epigenetic changes, genes, transcripts, and/or proteins matching the tailored list were included. Moreover, we retrieved relevant articles to MD and SV by inspecting the reference section of selected papers.

### Data Synthesis

Reference information, participant ancestry, study design, sample size, and participants’ age were extracted from studies that reported the selected genes or proteins in the “Results” section.

### Risk of Bias Assessment

ROBINS-E and SYRCLE tools [20, 21] were used to assess the risk of bias in non-randomized human and animal studies, respectively. A table was used to summarize the results using a color-coded scale.

Only six ROBINS-E domains were used: (1) confounding-induced bias, (2) bias in exposure measurement, (3) bias in participant selection for the study, (5) bias due to missing data, (6) bias in outcome measurement, and (7) bias in the selection of reported results. Domain (4) bias resulting from post-exposure interventions was excluded as it was irrelevant to this study. For animal and cellular models, items 3, 4, and 5 from SYRCLE were irrelevant and excluded from the analysis.

For studies where both human and animal were used, a mix of ROBINS-E and SYRCLE’s criteria was used.

### Hearing and Vestibular Phenotype in Knockout Mice of Stria Vascularis Genes

To explore the role of SV selected genes, auditory brainstem response and vestibular phenotype data were retrieved from the International Mouse Phenotyping Consortium (IMPC) portal for the selected genes in the existing mouse knockout database [22].

### MD Gene Expression in the Stria Vascularis

Gene expression data were retrieved from gEAR portal [23] from SV dataset [24] for all genes associated with Familial MD (*FAM136A*, *DTNA*, *PRKCB*, *COCH*, *DPT*, *SEMA3D*, *TECTA*, *GUSB*, *SLC6A7*, *HMX2*, *LSAMP*, *OTOG*, *STRC*, *MYO7A*, and *GJD3*) [15, 16]. A ± 1 logFC filter was used.

### Gene Set Enrichment Analysis

*GeneCodis* v4 [25] was used to perform Gene Set Enrichment Analysis (GSEA) using the overlapped genes between MD and SV employing functional annotations Gene Ontology (GO) Biological Process (BP), GO Cellular Component (CC), GO Molecular Function (MF) and KEGG Pathways, and regulatory annotation CollecTRI TFs.

## Results

**Fig. 1:**
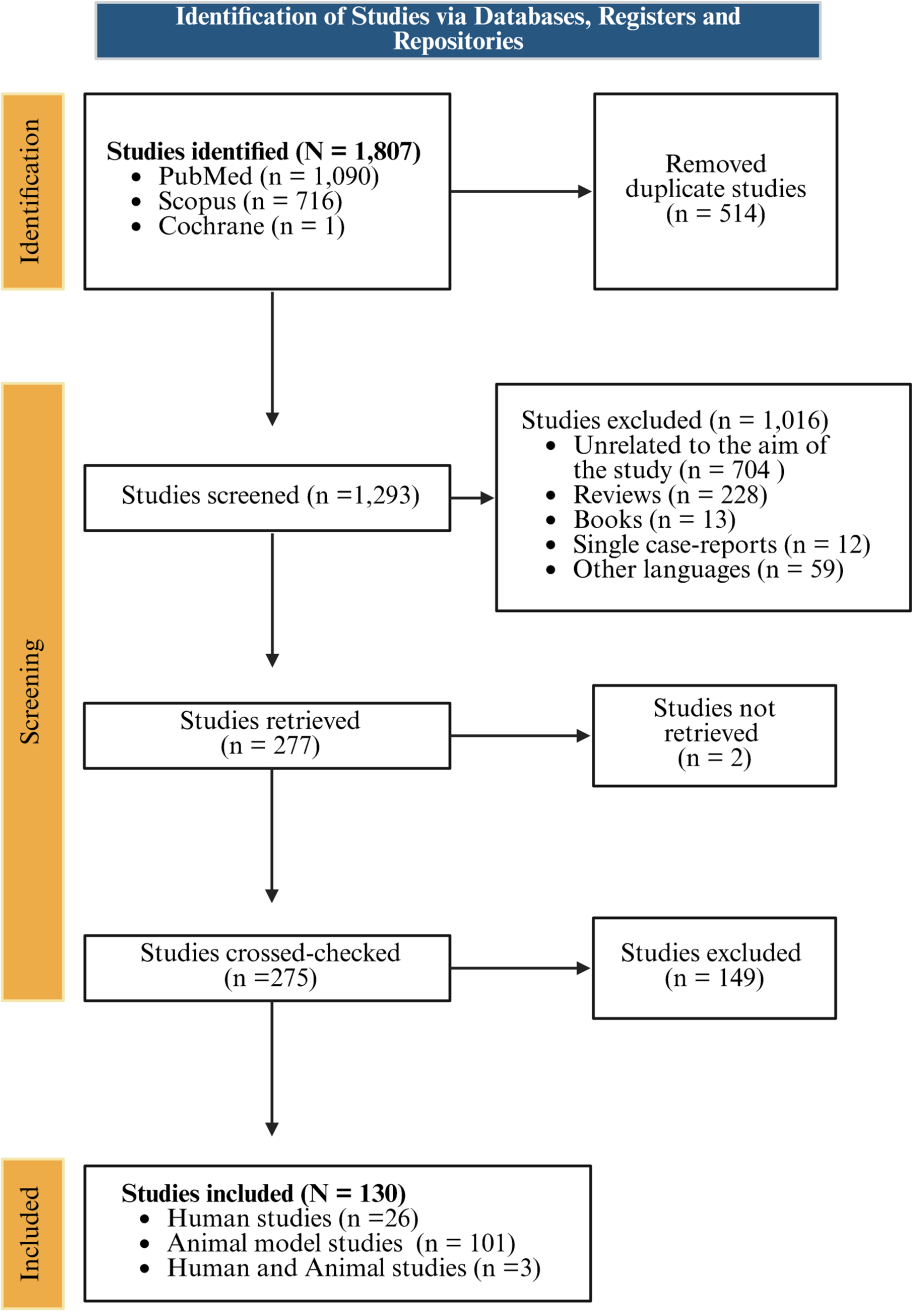
Flow diagram to select scientific articles from databases and registers for the systematic review

We selected 130 studies that fitted the inclusion criteria (Fig. 1).^1^ Of the total number of records, 26 were conducted on humans, and 101 were conducted on animals. Eighty were performed in mice (*Mus musculus*), 13 in rats (*Rattus norvegicus domestica*), five in guinea pigs (*Cavia* spp.), one in flies (*Drosophila melanogaster*), one in birds (*Gallus gallus domesticus* and *Tyto alba guttata*), one in frogs (*Xenopus laevis*), and one in gerbils (*Meriones unguiculatus*). Furthermore, three studies contained both human and animal subjects, one study used rats and mice, and one study used murine subjects and frogs (*X. laevis*).

### Human Studies

Out of the 26 articles conducted on humans, 15 had genes related to the SV (Table 1) [11, 26–39]. Seven studies were performed on MD, four studies including one on severe tinnitus, one on hereditary SNHL, one on endolymphatic sac, and one on hearing loss, and three did not explore any disease. Three of the studies were cross-sectional, and one was a retrospective, case–control study in silico analysis. None of the studies reported specific gene mutations; instead, they focused on mapping gene expression within the cochlea. These studies identified five genes on SV marginal cells—*HSPA4L*, *KCNE1*, *SLC12A2*, *PAX2*, and *HGF*; four on SV intermediate cells—*MYO5A*, *ACTB*, *KNCJ10*, and *EYA4*; nine genes on SV basal cells—*ACTN1*, *QSOX1*, *MDH1*, *COL11A2*, *ATP1A2*, *ATP1B3*, *SLC2A*, *CLDN11*, and *ABLIM3*; and four connexins—*GJB2*, *GJB6*, *GJA1*, and *GJE1*. Furthermore, *NRCAM*, *SORBS2*, *CACNA1D*, *ESRRB*, *ATP1B1*, *TYR*, and *LMX1A* were found to be markers for one or more SV cell types in the tailored list. Two studies also described three Familial MD genes: *OTOG*, *FAM136A*, and *PRKCB*.

**Table 1:** Summary of selected papers reporting genes in human subjects for Meniere disease (MD)

DOI	Author/Year	Country	Disease	Design	Sample Size	Sex/Median Age	Main Objective	Gene/Protein—List
10.1016/j.ebiom.2021.103309	Amanat, S. et al. (2021)	Spain	Severe tinnitus	Exome-based phenotype study	222	NA	To identify rare variants in synaptic genes in patients with severe tinnitus using whole-exome sequencing	*NRCAM*—Marginal/intermediate/basal cells
								*HSPA4LL*—Marginal cells
								*MYO5A*—Intermediate cells
10.1097/ONO.0000000000000027	Arambula, A. et al. (2023)	USA	MD	Case–control; in silico analysis; retrospective	NA	NA	To determine whether MD enriched perilymph proteins can be localized to specific cochlear cell types using single-cell and single-nucleus RNA-sequencing datasets	*NRCAM*—Marginal/intermediate/basal cells
								*ACTB*—Intermediate cells
								*ACTN1*—Basal cells
								*QSOX1; MDH1*—Marginal cells
10.1002/jcp.22737	Chiarella, G. et al. (2012)	Italy	MD	Proteomic study	20	Both/51 years old	To identify the possible biomarkers of MD using a proteomics-driven approach	*ACTB*—Intermediate cells
10.5152/iao.2019.5076	Dai, Q. et al. (2019)	China	MD	Comparative study	24	Both/30–43 years old	To investigate the gene polymorphisms in *Kcne1* and *Kcne3* in Familial and Sporadic MD patients compared to healthy controls in a Chinese cohort	*KCNE1*—Marginal cells
10.1159/000089410	Doi, K. et al. (2005)	Japan	MD	Genetic association study	505	Both/36–43 years old	To investigate whether specific single SNPs in the potassium channel genes *Kcne1* and *Kcne3* are linked to increased susceptibility in MD	*KCNE1*—Marginal cells
10.1186/s12864-024–10552-3	Fisch, KM. et al. (2024)	USA	MD	Cross-sectional study	1200	NA	To identify the rare and common genetic variants associated with unilateral MD using whole genome sequencing	*SORBS2*—Marginal/intermediate cells
								*CACNA1D*—Marginal/intermediate cells
								*COL11A2*—Basal cells
								*OTOG*—Familial MD
10.3389/fgene.2019.00076	Gallego-Martinez, A. et al. (2019)	Spain	MD	Targeted-sequence study	890	Both/NA	To assess the burden of rare variants of SNHL genes in Sporadic MD	*ESRRB*—Marginal/intermediate cells
								*FAM136A*—Familial MD
								*PRKCB*—Familial MD
								*GJB2*—Connexins
10.1007/s00441-010–0975-7	Ishiyama, G. et al. (2010)	USA	MD/Acoustic neuroma	Experimental study	18	Both/49–87 years old	To investigate the localization and expressions of aquaporins (Aqp1, Aqp4 and Aqp6) and proteins involved in endolymphatic homeostasis (Na⁺K⁺ATPase and Nkcc1) in MD patients	*SLC12A2*—Marginal cells
10.3389/fnmol.2022.973646	Liu, W. & Rask-Andersen, H. (2022)	Sweden	NA	Cross-sectional study	4	NA	To characterize the distribution of *Gjb2* and *Gjb6* gene transcripts in distinct cell types of the adult human cochlea using RNAscope® in situ hybridization	*KCNJ10*—Intermediate cells
								*CLDN11*—Basal cells
								*ATP1A2*—Basal cells
								*GJB2*—Connexins
								*GJB6*—Connexins
10.3389/fnmol.2022.857216	Liu, W. & Rask-Andersen, H. (2022)	Sweden	NA	Cross-sectional study	4	NA	To map the expression and cellular distribution of *Atp1a1*, *Atp1b1*, and *Atp1a3* gene transcripts encoding Na/K-ATPase isoforms in the adult human cochlea using RNAscope® in situ hybridization	*ATP1B1*—Marginal/intermediate/basal cells
								*ATP1B3*—Basal cells
								*GJB2*—Connexins
								*GJB6*—Connexins
10.1002/dneu.22279	Locher, H. et al. (2015)	The Netherlands; Belgium	Hereditary SNHL	Cross-sectional study	9	NA/W9-18	To investigate the embryological development of the human fetal stria vascularis between 9 and 18 weeks of gestation to characterize the expression key potassium-regulating and gap junction proteins involved in hereditary sensorineural hearing loss (SNHL)	*KCNQ1*—Marginal/intermediate cells
								*KCNJ10*—Intermediate cells
								*SLC2A1*—Basal cells
								*GJA1*—Connexins
								*GJE1*—Connexins
								*GJB2*—Connexins
								*GJB6*—Connexins
10.1007/s00441-019–03106-7	Nordström, CK. et al. (2020)	Sweden	Endolymphatic sac	Experimental study	NA	NA	To analyze Na/K-ATPase and its isoforms in the endolymphatic sac using super-resolution structured illumination microscopy	*ATP1B1*—Marginal/intermediate/basal cells
10.1002/dneu.22242	Pechriggl, EJ. et al. (2015)	Austria & Sweden	NA	Experimental study	24	NA/Embryos W8-12	To analyze the maturation and differentiation of neuronal markers in the fetal cochlea from gestational weeks 8–12	*PAX2*—Marginal cells
10.1016/j.ajhg.2022.04.010	Trpchevska, N. et al. (2022)	Various	Hearing Loss	Genetic association and meta-analysis study	723,266	Both/60 years old	To perform a genome-wide association meta-analysis in hearing loss patients to identify the risk loci in cochlear structures	*TYR*—Marginal/intermediate/basal cells
								*LMX1A*—Marginal/intermediate cells
								*EYA4*—Intermediate cells
								*ABLIM3*—Basal cells
								*GJB2*—Connexins
								*GJB6*—Connexins
10.1038/s41598-022–20774-8	Zou, J. et al. (2022)	China & Finland	MD	Case–control study	77	Both/36–54 years old	To determine the cytokine profiles of MD and its potential role in autoimmune/autoinflammatory mechanisms	*HGF*—Marginal cells

In the retrieved studies, *GJA1*, *GJB2*, and *GJB6* were found to be expressed in spiral ligament fibrocytes. Interestingly, *GJE1* (known as connexin 23 in mice) was the only connexin identified in the SV, localized in the basolateral membrane of the future marginal cells. However, by embryonic day 16, *GJE1* expression was downregulated in marginal cells and was found only in adjacent cochlear cells.

Upon reviewing the reference list of these four records, we found one publication [40] that described three relevant genes to MD and SV; one was expressed in basal cells—*GSTM1*—and two found in intermediate cells—*TMEM176A* and *TMEM176B.* These three genes were found to be related to the immune response in Sporadic MD.

### Joint Human and Animal Studies

Both studies involving human and animal subjects (Table 2) were conducted in humans and mice [41–43]. The only gene identified in the two studies was *KCNJ10*, expressed in SV intermediate cells. Neither of the two studies were related to MD, although they investigated hearing loss and particularly age-related hearing loss (ARHL).

**Table 2:** Summary of selected papers reporting genes in humans and mice (*Mus musculus*)

DOI	Author/Year	Country	Disease	Design	Sample Size	Sex/Median Age	Main Objective	Gene/Protein - List
Human - Mice (*Mus musculus*)								
10.1016/j.heares.2024.109091	Chen, J. et al. (2024)	UK	Hearing Loss	Experimental; Case-control	Mice: 3–7; Human: 6099	Mice: Both sexes/~14 weeks; Human: NA/44–45 years	To investigate how *Sgms1* deficiency leads to progressive hearing loss, focusing on endocochlear potential and stria vascularis pathology.	*Kcnj10* - Intermediate cells
10.1113/jphysiol.2006.116889	Knipper, M. et al. (2006)	Germany	Progressive High-Frequency Hearing Loss	Mouse Model	4-6 cochleae per age group	NA/NA	To study *Limp2's* role in progressive high-frequency hearing loss via *Kcnq1*/*Kcne1* and megalin loss in the stria vascularis.	*Kcnq1* - Marginal/intermidate cells
								*Kcne1* - Marginal cells
								*Kcnj10 *- Intermidate cells
10.1016/j.neurobiolaging.2019.04.009	Liu, T. et al. (2019)	China & USA	ARHL	Experimental; Case-only	Mice:NA; Human: 12	Mice: Sexes/1.5-month-old to 2.5 years old; Human: Both sexes/30 to 91 years old.	To determine if age-related degeneration of neural crest–derived nonsensory cells in the stria vascularis, outer sulcus cells, and satellite cells in the spiral ganglion, contribute to the development of metabolic and neural forms of age-related hearing loss	*Kcnj10* - Intermediate cells

### Animal Studies

Of the 80 mouse model studies analyzed, 52 reported genes expressed in the SV list (Supplementary Table S2). Only seven studies identified Familial MD genes alongside SV marker genes [24, 44–49]. These included two studies on ARHL and one study each on human deafness and head bobbing, hearing impairment, hypothyroidism-related SNHL, hearing loss, and one with no disease associated. The reported Familial MD genes were *Myo7a*, *Hmx2*, *Otog*, *Tecta*, and *Coch*. The SV intermediate cell maker *Kcnj10* was reported in several studies. In contrast, marginal cell marker genes were described only once—*Hspa1b* and *Atp1b2*. Similarly, intermediate cell marker genes—*Hsp90b1*, *Hspa5*, and *Rorb*—and basal cell marker genes—*Skp1*, *Spa1a*, *Cebpb*, *S100b*, *Cdkn1a*, *Nudt4*, and *Slc2a1*—were reported only once. Furthermore, *Pde4b* and *Kcnq1* were reported in more than one cell marker in the tailored list. *Gjb2* was reported once, with expression in cochlear supporting cells and SV basal cells.

Ten rat studies reported genes in the SV (Table 3) [50–59], among them *Atp1b2*, *Slc12a2*, *Slc2a13*, and *Kcne1* in the marginal cells; *Kcnj10*, *Slc45a2*, and *Hif1a* in the intermediate cells; and *Anxa5*, *Lamp1*, *Slc2a1*, *Mt1a*, *Arp1a2*, and *Cldn11* in the basal cells. *Cacna1d*, *Kcnq1*, *Kcnj13*, *Atp1b1*, and *Atp2b1* were reported in two or more SV cell types. In the retrieved studies, *Gjb2* was reported in type 1 fibrocytes. None of the studies focused on a particular disease. Similarly, five guinea pig studies found genes *Atp1b2* and *Slc12a2* in marginal cells, *Kcnj10* in intermediate cells, and *Rapgef3* and *Cldn11* in basal cells, and the described connexins were *Gjb2*, *Gjb3*, and *Gjb6*. Additionally, *Pax3* and *Kcnq1* were reported as markers for two or more cell types (Table 4) [60–64]. In the retrieved records, *Gjb2* was expressed in the basal cells at embryonic day 40 (E40); from E45-E50, it is absent from the cochlea, and from E60 onwards, the expression is found in fibrocytes and spiral ligament. *Gjb3* and *Gjb6* had no change in expression in the lateral wall.

**Table 3:** Summary of selected papers reporting genes in rats (*Rattus norvegicus domestica*).

DOI	Author/Year	Country	Disease	Design	Sample Size	Sex/Median Age	Main Objective	Gene/Protein - List
*Rattus norvegicus domestica*								
10.1093/abbs/gms024	Chen, J. et al. (2012)	China	NA	Experimental Study	6	NA/P3-5	To investigate the distribution and expression of Cav1.3 channels in the rat cochlea	*Cacna1d* - Marginal/Intermediate Cells
10.1007/s10571-014-0036-y	Gross, J. et al. (2014)	Germany	NA	Cellular Model	NA	NA/P3-5	To compare the expression of genes involved in different cell death patterns in the stria vascularis, organ of Corti and the modiolus.	*Hif1a *- Intermediate Cells
10.1016/j.biocel.2022.106259	Huang, S. et al. (2022)	China	NA	Cellular model	NA	NA/NA	To investigate the role of HIF-1α-mediated autophagy in hypoxic cochlear marginal cells and its protective mechanism against apoptosis	*Hif1a *- Intermediate Cells
								*Anxa5* - Basal Cells
10.1080/03655230701624830	Gu Hur, D. et al. (2007)	Korea	NA	Experimental Study	12	Female/W7	To determine whether skeletal muscle denervation causes post-transcriptional mechanisms to regulate AChR β-subunit mRNAs.	*Kcnq1 *- Marginal/Intermediate Cells
								*Kcne1 *- Marginal Cells
10.1038/srep20903	Liu, J. et al. (2016)	China	NA	Cellular model	NA	NA/NA	To identify the cell organelles that store ATP in cochlear marginal cells	*Lamp1* - Basal Cells
10.1016/j.heares.2011.03.011	Mazurek, B. et al. (2011)	Germany	NA	Cellular model	18	NA/P3-P5	To investigate the expression of genes involved in oxidative stress response in cochlear tissues and to explore their role in cochlear injury during the developing period under different oxygen conditions.	*Slc2a1* - Basal Cells
								*Mt1a* - Basal Cells
10.1038/s41598-018-38079-0	Nonomura, Y. et al. (2019)	Japan	NA	Experimental Study	64	Male/W7	To profile and structurally characterise N-glycans in the stria vascularis to see how they are involved in regulating membrane activity and secreted proteins.	*Cldn11 *- Basal Cells
10.1152/physiolgenomics.00006.2005	Pondugula, S.R. et al. (2006)	USA	NA	Cellular model	NA	NA/NA	To identify the genes involved in Na⁺ transport in rat semicircular canal duct epithelium and determine how their expression is regulated by glucocorticoids.	*Kcnj13 *- Marginal/Intermediate Cells
								*Kcnj10* - Intermediate Cells
10.1016/j.neures.2013.06.003	Takiguchi, Y. et al. (2013)	Japan	NA	Histological; immunohistochemical	12	NA/W6-W8	To investigate the long-term effects of mitochondrial dysfunction induced by 3-nitropropionic acid on cochlear fibrocytes and ion transport proteins in the cochlea, and examine changes in the expression of key proteins in response to cochlear degeneration	*Slc12a2 *- Marginal Cells
								*Kcnj10* - Intermediate Cells
								*Gjb2* - Connexins
10.1111/ejn.12973	Uetsuka, S. et al. (2015)	Japan	NA	Experimental	109	NA/W7	To identify proteins involved in ion and organic substance transport in the stria vascularis and discover new candidates for deafness-related genes.	*Atp1b1 *- Marginal/Intermediate/Basal Cells
								*Kcnj13* - Marginal/Intermediate Cells
								*Atp2b1* - Marginal/Intermediate Cells
								*Kcnq1* - Marginal/Intermediate Cells
								*Kcne1* - Marginal Cells
								*Slc2a13 *- Marginal Cells
								*Slc12a2* - Marginal Cells
								*Atp1b2* - Marginal Cells
								*Slc45a2* - Intermediate Cells
								*Kcnj10* - Intermediate Cells
								*Slc2a1* - Basal Cells
								*Atp1a2* - Basal Cells

**Table 4:** Summary of selected papers reporting genes in guinea pigs (*Cavia* spp.), birds (*Gallus gallus domesticus* and *Tyto alba guttata*) and frogs (*Xenopus laevis*)

DOI	Author/Year	Country	Disease	Design	Sample Size	Sex/Median Age	Main Objective	Gene/Protein—List
*Cavia* spp.								
10.3389/fnmol.2022.842132	Edvardsson Rasmussen, J. et al. (2022)	Sweden	NA	Experimental	18	Both/W6-9	To investigate the time-dependent effects of acute furosemide administration by localizing Fetuin-A and PEDF proteins in the guinea pig cochlea	*Slc12a2*—Marginal cells
10.1007/s00441-006–0369-z	Jin, Z, et al. (2007)	Sweden	Hereditary deafness	Experimental	NA	NA/varied from E35 to adult	To investigate the molecular basis of hereditary deafness in the German waltzing guinea pig	*Pax3*—Marginal/intermediate cells
								*Atp1b2*—Marginal cells
								*Kcnj10*—Intermediate cells
								*Gjb2*—Connexins
								*Gjb3*—Connexins
								*Gjb6*—Connexins
10.1111/j.1460–9568.2007.05994.x	Jin, Z, et al. (2008)	Sweden	Hereditary deafness	Experimental	NA	NA/E35-P0	To examine the developmental patterns of key potassium transport proteins in the cochlear lateral wall of German waltzing guinea pigs with hereditary deafness	*Slc12a2*—Marginal cells
								*Cldn11*—Basal cells
10.1007/s00405-022–07380-0	Wang, C. et al. (2022)	China	NA	Experimental	30	NA/NA	To investigate the expression and localization of *Epac1* and *Epac2* in the guinea pig inner ear and their potential role in inner ear microcirculation	*Rapgef3*—Basal cells
10.1007/s00405-014–3093-4	Xiong, M. et al. (2014)	China	NA	Experimental	30	NA/NA	To determine whether Radix astragali can prevent or reduce the down-regulation of connexin 26 in the Stria vascularis of the guinea pig cochlea following acoustic trauma	*Kcnq1*—Marginal/intermediate cells
*Gallus gallus domesticus* & *Tyto alba guttata*								
10.1038/srep34203	Wilms, V. et al. (2016)	Germany	NA	Experimental	NA	NA/varied from P18 to P966	To investigate the molecular bases of K + secretory cells in the inner ear in birds and mammals	*Kcnj10*—Intermediate cells
								*Atp1a2*—Basal cells
								*Gjb2*—Connexins
								*Gjb6*—Connexins
*Xenopus laevis*								
10.1159/000356353	Parrock, S. et al. (2013)	UK	EAST Syndrome	Case-only	NA	NA/NA	To characterize *Kcnj10* mutations in patients with EAST syndrome to investigate the role of *Kcnj16* co-expression in modulating *Kcnj10* channel function	*Kcnj10*—Intermediate Cells

On the other hand, one study performed on birds had genes *KCNJ10* and *ATP1A2* from intermediate and basal cells, respectively, and the described connexins—*GJB2* and *GJB6*—were not found expressed in the SV [65]. The paper that described several connexins in the guinea pig was focused on hereditary deafness, whereas the study that utilized birds as a model was performed in wild-type animals. The *X. laevis* study only identified the intermediate cell gene *KCNJ10* while investigating Epilepsy, Ataxia, Sensorineural deafness, and Tubulopathy (EAST) syndrome [66].

### Risk of Bias Assessment

Human and animal risk of bias analyses are summarized in Supplementary Tables S4 – S6.

### Audiovestibular and Immune Phenotype in Mouse Knockout of Stria Vascularis Genes

Knocked out *Gja1* generated mice had significant immune dysregulation with an abnormal spleen morphology. Furthermore, *Kcne1*-knockout mice had an increased number of monocytes and neutrophils and a decreased number of lymphocytes, as well as severe hearing loss across all frequencies (Table 5).

**Table 5:** Audiovestibular and immune response phenotype information from the International Mice Phenotyping Consortium for the selected SV genes

Gene	Audiovestibular	Immune response
*Actn1*	NS	NS
*Qsox1*	NS	NS
*Mdh1*	NS	NS
*Actb*	–	–
*Kcne1*	Significant	Significant
*Esrrb*	–	–
*Cacna1d*	–	–
*Coll11a2*	NS	NS
*Slc12a2*	–	–
*Hgf*	–	–
*Gstm1*	–	–
*Gjb2*	–	–
*Gjb6*	–	NS
*Gja1*	NS	Significant
*Gje1*	NS	NS
*Tmem176a*	NS	–
*Tmem176b*	–	–

*NS* not significant

### Familiar MD Gene Expression in the Stria Vascularis

Three single-cell preservation methods from the lateral wall dataset in the gEAR portal: fresh tissue, RNA-later, and methanol fix, were used. The only gene that was found consistently across all three preservation methods was *Coch*, which had high expression in fibrocytes with 2.08 logFC and present in 63% of cells (fresh tissue), 1.78 logFC and found in 70% of cells (RNA-later), 1.41 logFC and 65% of cells (methanol fix) (Table 6). On the other hand, *Dtna* was found highly expressed in fresh tissue Reissner’s cells (1.10 logFC in 49% of cells) and in RNA-later spindle cells (1.05 logFC in 70% of cells). *Prkcb* was found in immune cells: B cells in fresh tissue (1.23 logFC in 51% of cells) and macrophages in RNA-later and methanol fix (1.00 logFC in 60% of cells and 1.06 logFC in 68% of cells, respectively). Furthermore, *Gjd3* was not found in any of the cells in the dataset.

**Table 6:** Average expression of Familial MD genes in the lateral wall from Gu et al. (2020) found in the gEAR portal

Method	Spindle/root	Fibrocytes	Reissner’s	B cells	Macrophages
Fresh tissue	–	*Coch* (*N* = 30, 63.33%, mean: 2.08)	*Dtna* (*N* = 51, 49.02%, mean: 1.10)	*Prkcb* (*N* = 142, 51.41%, mean: 1.23)	–
RNA-later	*Dtna* (*N* = 54, 70.37%, mean: 1.05)	*Coch* (*N* = 74, 70.21%, mean: 1.78)	–	–	*Prkcb* (*N* = 15, 60.00%, mean: 1.00)
Methanol fix	–	*Coch* (*N* = 101, 65.35%, mean: 1.41)	–	–	*Prkcb* (*N* = 25, 68.00%, mean: 1.06)

### Gene Set Enrichment Analysis

**Fig. 2:**
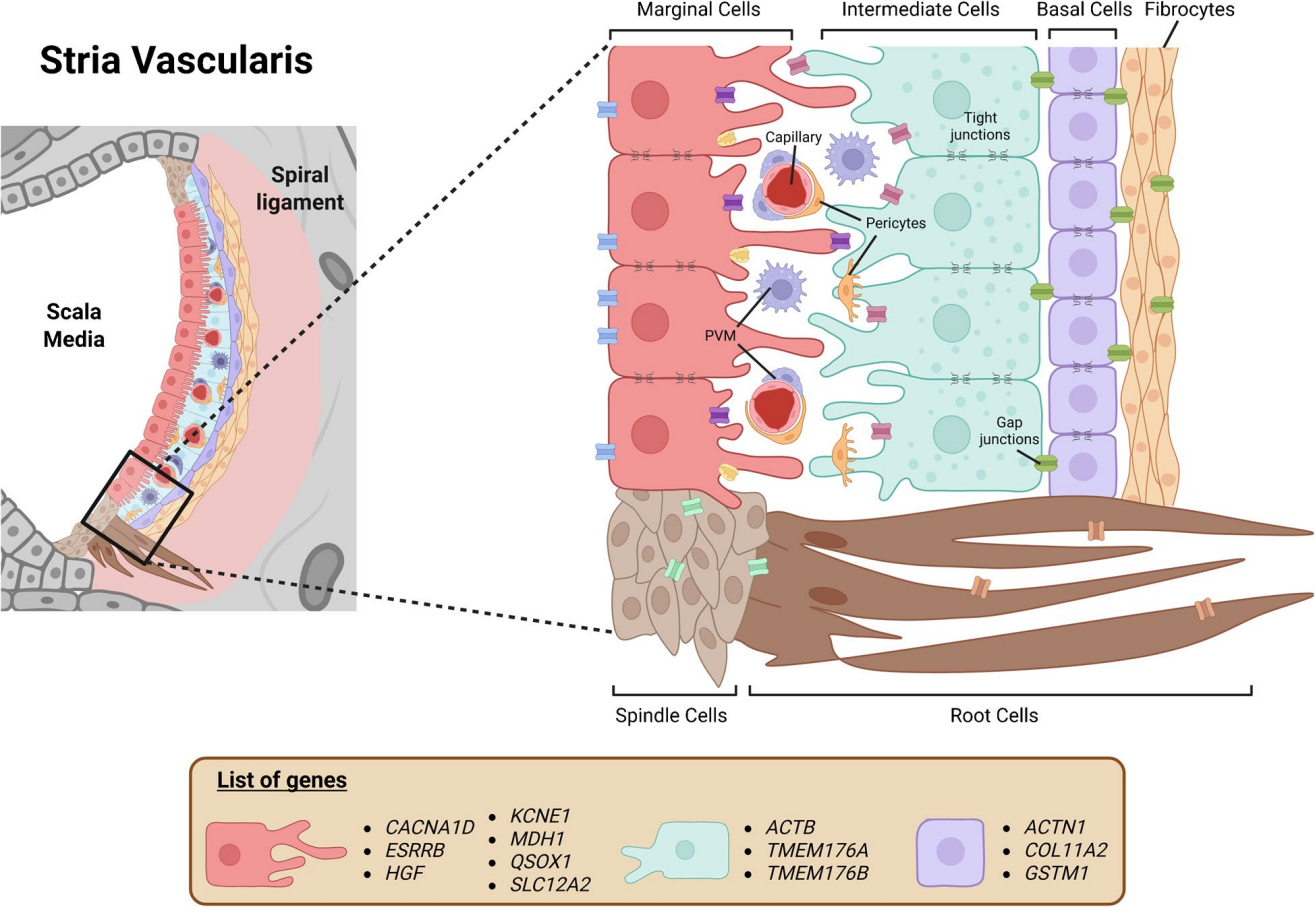
Representation of the stria vascularis (SV) and MD genes found in the lateral wall of the scala media

Gene Set Enrichment Analysis was performed using “human” as species on candidate genes: *ACTN1*, *QSOX1*, *MDH1*, *ACTB*, *KCNE1*, *ESRRB*, *CACNA1D*, *COL11A2*, *SLC12A2*, and *HGF* (Fig. 2),^2^ and connexins *GJA1*, *GJB2*, *GJB6*, and *GJE1*. The resulting active pathways were not specific to the inner ear auditory and vestibular dysfunction, nor immune dysregulation. The top three enriched pathways for GO BP (Supplementary Table S7) were “cell communication by electrical coupling” (adjusted *p*-value [adjusted *p*-val] = 1.61 × 10^−6^; Relative Enrichment [RE] = 558), “maintenance of blood–brain barrier” (adjusted* p*-val = 1.61 × 10^−6^; RE = 153), and “gap junction assembly” (adjusted* p*-val = 1.71 × 10^−6^; RE = 488). For GO CC (Supplementary Table S8) and GO MF (Supplementary Table S9), the top two enriched pathways related to gap junction activity were “connexin complex” (adjusted* p*-val = 1.11 × 10^−7^ RE = 260), and “gap junction” (adjusted* p*-val = 7.17 × 10^−5^ RE = 132), and “gap junction channel activity” (adjusted* p*-val = 7.57 × 10^−8^; RE = 238), and “gap junction channel activity involved in cell communication by electrical coupling” (adjusted* p*-val = 7.57 × 10^−8^; RE = 982).

For KEGG (Supplementary Table S10), the three principal pathways were “arrhythmogenic right ventricular cardiomyopathy” (adjusted* p*-val = 1.05 × 10^−2^; RE = 29), “Focal adhesion” (adjusted* p*-val = 3.35 × 10^−2^; RE = 12) and “Tight junction” (adjusted* p*-val = 3.35 × 10^−2^; RE = 15).

No transcription factors (TF) were significant (Supplementary Table S11). It is worth noting that the gene count was always below 50% in the GO, KEGG, or collecTRI analyses.

## Discussion

Menière’s Disease is a multifactorial disease with genetic contribution in 20–30% of cases [15]. In this review, we analyzed studies published over the past 20 years that investigated gene expressions in SV marginal, intermediate, and basal cells, and the relevance to MD. These studies included both human and animal models and described over 20 genes in total. Among these, 13 SV-associated genes—*ACTB*, *ACTN1*, *CACNA1D*, *COL11A2*, *ESRRB*, *GSTM1*, *HGF*, *KCNE1*, *MDH1*, *QSOX1*, *SLC12A2*, *TMEM176A*, and *TMEM176B*—and gap junction proteins were consistently identified as being particularly important in the context of MD pathogenesis.

### Marginal Cells

Marginal cells are the innermost cell type of the SV and are in direct contact with the scala media [67]. Their primary function is the active transport of potassium ions (K^+^) from the other outer SV cell types to cochlear duct to maintain endocochlear potential [68]. We found that marginal cell markers *QSOX1* and *MDH1* were reported in two independent MD studies [38, 40]. The sulfhydryl oxidase 1 protein (*QSOX1* gene) is a crucial disulfide bond-forming enzyme that facilitates integration of laminin into the extracellular matrix, for the matrix ability to support integrin-dependent cell adhesion and migration [69]. Although the role of *QSOX1* encoded protein in audiovestibular dysfunction is yet to be elucidated, studies have found that *QSOX1* may be involved in the immune response exerting anti-inflammatory effects in sepsis, by inhibiting M1 macrophage polarization. Furthermore, it regulates inflammatory signaling by preventing epidermal growth factor receptor activation and promoting ubiquitin-mediated regulation [70]. On the other hand, malate dehydrogenase, cytoplasmic protein (*MDH1* gene) is a NAD(H)-dependent enzyme [71], and evidence of its function on the inner ear is scarce. The *MDH1*-encoded protein is part of the malate-aspartate shuttle (MAS), crucial to maintaining intracellular NAD(H) redox balance by transferring reduced equivalents across the impermeable inner mitochondrial membrane, as NAD(H) cannot cross it [72]. A homozygous variant in *MDH1* (p.Ala138Val) has been linked to developmental delay, epilepsy, and progressive microcephaly [73]. Conversely, *MDH1* can drive functional impairment to CD^8+^ T cells in patients with triple-negative breast cancer, promoting their differentiation to a CD^8+^ T exhausted phenotype, which may disrupt immune balance within the breast cancer environment [74].

The potassium voltage-gated channel subfamily E member 1 protein, encoded by *KCNE1* gene, functions by dimerizing with *KCNQ1*-encoded protein to modulate the channels K^+^ secretion to the cochlear and vestibular endolymph [75–77]. Homozygous or compound heterozygous variants in the *KCNE1* and *KCNQ1* have been associated with the Jervell and Lange-Nielsen syndrome type 2 [78–80], characterized by cardiac arrhythmias and increased risk of sudden death, and associated with severe bilateral sensorineural hearing loss. In the tailored list, both *ESRRB* and *CACNA1D* were found to be upregulated in marginal cells but downregulated in intermediate cells. *ESRRB* encodes the steroid hormone receptor ERR2 protein, and *CACNA1D* encodes the voltage-dependent L-type calcium channel subunit alpha-1D. Both proteins have been implicated in hearing loss–related disorders, such as DFNB35 and familial sinus node dysfunction [81–85]. However, none of the cases with these disorders have presented vestibular dysfunction, commonly observed in MD patients.

Solute carrier family 12 member 2 protein is encoded by *SLC12A2* gene. Its main function is to transport Cl^−^, K^+^, and/or Na^2+^ across the plasma membrane [86]. Research on the stria vascularis has reported that variants in the carboxy-terminal domain of *SLC12A2*-encoded protein are responsible for hereditary hearing loss across all frequencies. The study also reported that some of the patients with these mutations had vestibular impairments [87]. Furthermore, *SLC12A2* has been implicated in the immune system, being critical for apoptotic cell uptake. The study found that deficiency of *SLC12A2* increased efferocytosis in phagocytes and macrophages, exacerbating the inflammatory response [88].

*HGF* gene encodes for hepatocyte growth factor, and it has been linked directly to the immune response in MD cases [6, 37]. Hepatocyte growth factor receptor is encoded by the *MET* gene, and it is expressed in the cell’s surface [89]. HGF/MET signaling has been involved in immunoregulating processes such as monocyte-macrophage polarization, dendritic cell migration and deactivation, and B cell adhesion [89–92]. In the context of MD, the *HGF*-encoded protein—along with granulocyte colony-stimulating factor (G-CSF) and IL-8—has been hypothesized to contribute to the formation of neutrophil extracellular traps, contributing to the inflammatory response [37]. Furthermore, a single-cell RNA sequencing study found that patients with an autoinflammatory MD phenotype exhibit an active population of monocytes characterized by high transcript levels of IL-1β receptors and IL-8. The study found that the monocytes could be polarized by a combination of HGF, vascular endothelial growth factor (VEGF), and stem cell factor (SCF) [6]. Besides its involvement in the immune system, it has been reported that noncoding mutations in hepatocyte growth factor have been associated with DFNB39 [93].

### Intermediate Cells

*ACTB* encodes protein actin, cytoplasmic 1, also known as beta-actin. Beta-actin is an important structural protein, and it is expressed in almost all adult human cell types [94, 95]. Mutations in *ACTB*-encoded protein have been linked to an array of diseases ranging from deafness to lymphomas [96, 97]. Several studies have found that mutations in the *ACTB* gene lead to the Dystonia-Deafness syndrome, characterized by sensorineural hearing loss and dystonia [96, 98].

Intermediate cells are neural crest-derived melanocytes that form the middle layer of the SV [99]. They contribute to the supporting function of the cochlear blood labyrinth barrier (BLB), which strictly regulates the flow of ions and metabolites from the capillary network to the endolymph [99]. There is a link to congenital deafness in intermediate cells, as the deficiency of these cells results in a low endocochlear potential that is insufficient to reach the threshold of compound action potential through sound pressure [100]. In a previous study, researchers were able to identify marker genes *TMEM176A* and *TMEM176B*, which are associated with the intermediate cells of the SV and are also associated with MD [40, 101]*.* Most of the studies examined the co-regulation and interaction of *TMEM176A* and *TMEM176B*, given their structural similarities and shared involvement in intracellular dynamics. Transmembrane protein 176 A (*TMEM176A* gene) and Transmembrane protein 176B (*TMEM176B* gene) are homologous genes that suppress the maturation of dendritic cells [102]. Although in vivo studies of these genes remain largely unknown, previous knockout mouse models found that *TMEM176A/B* is functionally involved in the MHC class II presentation by regulating the type-2 conventional dendritic cells (cDC2) [103]. Furthermore, the proteins encoded by *TMEM176A/B* localize in the Golgi apparatus in the late endolysosomal system and colocalize with HLA-DM, which catalyzes peptide exchange on MHC class II molecules [103, 104]. Although there are limited studies that cater *TMEM176A* functionality, *TMEM176B* in particular has also been known to mediate Na + efflux for facilitating charge compensation for V-ATPase driven acidification [102, 105]. This process regulates phagosomal pH, which is critical for antigen cross-presentation to dendritic cells [105]. Additionally, other knockout studies of *TMEM176B* have found defective functionality in cerebellar granulocytes, which resulted in the development of sporadic ataxia [102].

### Basal Cells

Basal cells are the outermost layer of the SV that forms a critical barrier between the endolymph and intrastrial space [100]. They connect to the fibrocytes of the spiral ligament, allowing them to regulate ion exchange into the SV while preventing leakage between cochlear compartments [99]. In the study, we identified *GSTM1* and *ACTN1* as basal cell markers for the SV. Glutathione S-transferase Mu 1 (*GSTM1* gene) is a detoxification enzyme that catalyzes the conjugation of reduced glutathione (GSH) to electrophilic compounds [106]. They also serve a protective role against carcinogens such as polycyclic aromatic hydrocarbons (PAH), suggesting that the deficiency of this gene increases the risk of cancer [107]. Aside from xenobiotic metabolism, *GSTM1* is involved in modulating cell signaling by conjugating prostaglandins A_2_ and J_2_ (PGA_2_ and PGJ_2_, respectively). This enzymic activity mitigates the antiproliferative effects of these prostaglandins, suggesting that it may play a role in cell proliferation and inflammatory process [108]. Additionally, *GSTM1* modifies lipid signaling molecules by catalyzing 14,15-Hepoxilin A_3_ (14,15-HxA_3_) to a cysteinyl derivative (14,15-HxA_3_) [109]. On the other hand, Alpha-actinin-1 (*ACTN1* gene) is a cross-linking protein for filamentous actin (F-actin) that contributes to cytoskeletal organization and cell adhesion [110]. Abundantly expressed in the smooth muscle cells, *ACTN1* is essential for T-cell migration and functionally interacts with ICAM-1 to facilitate leukocyte migration to the surrounding tissues [111]. It also binds to CLP36, forming a complex that associates with actin filaments and stress fibers in activated platelets and endothelial cells [112]. *ACTN1* is found in the stereocilia of hair cells where it plays a critical role in actin-binding activity that is regulated by Ca^2+^ through MET channels [113, 114]. Although it is expressed at low levels in the auditory and vestibular stereocilia, this process forms an essential part of stabilizing the stereocilia cytoskeleton [113, 114].

Collagen alpha-2(XI) chain is encoded by the *COL11A2* gene. The role of *COL11A2* in the inner ear is not well known; however, several collagen proteins are expressed within the cochlea [115]. A study on Zebrafish identified that *COL11A2* is essential in maintaining the properties of the cartilage matrix [116]. Several studies have reported that mutations in the *COL11A2* gene lead to non-syndromic and syndromic deafness [117, 118]. Research has shown that mutations in *COL11A2* can lead to syndromes that may have concurrent vestibular impairments, such as Stickler Syndrome [119].

### Connexins

Gap junction proteins are a protein family essential in epithelial intracellular junctions [120], as they form hemichannels in the plasma membrane from hexameric structures, known as a connexon [121]. The union of two connexins between two cells is known as a gap junction, which allows for intracellular exchange of molecules, such as Ca^2+^, ATP, glutamate, or NAD^+^ [122]. In this review, we found two connexins expressed in the SV—*GJB2* and *GJE1*. The gap junction beta-2 protein (*GJB2* gene) has been identified in non-syndromic SNHL, with three main mutations c.35delG driving 40–70% of deafness cases in the European, North African, Middle Eastern, Sub-Saharan African, North and South American, and Australian populations [123, 124]. On the other hand, c.235delC and c.109G > A variants are more frequently found across several East Asian populations [121]. Gap junction epsilon-1 protein (*GJE1* gene) role in hearing loss is less studied. A study found 13 (5.14%) of 253 unrelated Taiwanese patients with nonsyndromic hearing loss had variants in *GJE1* [125]. Although *GJB2* and *GJE1* were the only two connexins after filtering, *GJA1*, *GJB3*, and *GJB6* were present in some of the selected studies.

Gap junction alpha-1 protein (*Gja1* gene) knockout mice, generated by the IMPC, were found with abnormal spleen morphology. Although no audiovestibular phenotype was recorded by the IMPC, the *GJA1*-encoded protein is essential in humans, and mutations in the gene have been found to contribute to nonsyndromic hearing loss [126]. Furthermore, a study found that suppressing *Gja1* in mice creates hyperpermeability in the SV, decreasing endocochlear potential, leading to mild hearing loss [127].

The importance of connexins in MD has yet to be fully elucidated; however, several variants forming a haplotype in gap junction delta-3 protein (*GJD3* gene) have been found in both sporadic and Familial MD, suggesting that rare variation in connexins can lead to audiovestibular dysfunction [16].

### Stria Vascularis and Menière’s Disease

Besides the SV marker genes used in this study to screen previously published papers, an independent study found 181 genes enriched in Sporadic MD and 264 genes in Familial MD, with 43 genes shared between both MD subsets [12], among them the *KIF1B* gene. A variant in the *KIF1B* gene has been found to possibly contribute to pathogenesis in the autoinflammatory phenotype in some Sporadic MD patients [128]. None of the Familial MD genes showed to be enriched in the single-cell RNA sequencing SV dataset, although we did find enrichment of *Prkcb* in B cells and macrophages. A mutation in a conservative serine phosphorylation site in the protein kinase C beta type (*PRKCB* gene) has been associated with increased tyrosine phosphorylation and membrane association of Bruton's tyrosine kinase and increased signaling through the B-cell receptor (BCR) in B cells and the high-affinity IgE receptor in mast cells. Furthermore, *PRKCB* inhibition causes an overexpression of Ca^2+^ signaling triggered by BCR [129].

A study published in 2025 using single-cell RNA sequencing on SV whole-tissue explants from neonatal and mature mice found that TNF-like Weak inducer of apoptosis (TWEAK) was released from the intermediate cells of the SV to the marginal cells and spindle cells, where its receptor tumor necrosis factor receptor superfamily member 12 A (*TNFRSF12A* gene) was expressed [18]. Additionally, a study on MD reported that patients with the allelic variant rs4947296 (chr6:31014645T > C) showed 973 differentially expressed genes, including NF-kappa-B p105 subunit *(NFKB1* gene) and *TNFRSF12A* [130]. Furthermore, carriers of the CC risk genotype had higher expression of both *TNFRSF12A* and *NFKB1* compared to the TT protective genotype. When haplotype-conditioned lymphocytes were stimulated with TWEAK, the CC genotype had no significant increase in expression of *TNFRSF12A*; conversely, *NFKB1* was significantly upregulated when compared to the stimulated TT genotype. This allelic variant is an expression quantitative trait locus that regulates the TWEAK pathway, leading to the activation of NF-κB inflammatory pathway in MD patients [130].

## Limitations

This systematic review has its limitations. First, the SV marker gene list was generated from P30 mice and described MD genes in humans. Second, the number of SV studies in MD patients or animal models is low. Third, most of the studies lacked information on genetic variants and their effects on the inner ear. Additionally, some of the studies exhibited moderate to high risk of bias; thus, results should be interpreted with caution. Finally, while animal models were included, protein function often differs across species, limiting the extension of findings to MD patients.

## Conclusions

Some genes found in the SV may contribute to the auditory dysfunction in MD, but most genetic variants may be involved in the immune response commonly found in Sporadic MD cases. The role of gap junction proteins in the SV and their connection to MD pathophysiology is yet to be elucidated.

## Supplementary Information

Below is the link to the electronic supplementary material.

ESM 1 (XLSX 118 KB)

## Data Availability

The code used to retrieve gene and protein names can be found in https://github.com/MDNLAtlas/UniProt_data_parser.git.

## Footnotes

1 Created in BioRender. Lopez-Escamez, J. (2025) https://biorender.com/ah8dnhm

2 Created in BioRender. Lopez-Escamez, J. (2025) https://BioRender.com/93kcm0f
